# The influence of organisational climate on care of patients with schizophrenia: a qualitative analysis of health care professionals’ views

**DOI:** 10.1007/s11096-016-0247-z

**Published:** 2016-01-21

**Authors:** Jane Sutton, Hannah E. Family, Jennifer A. Scott, Heather Gage, Denise A. Taylor

**Affiliations:** University of Bath, Bath, UK; University of Surrey, Guildford, UK

**Keywords:** Clozapine, Medication management, NHS, Organizational change, Organizational climate, Pharmacist prescriber, Philosophy of care, Role ambiguity, Schizophrenia, United Kingdom

## Abstract

*Background* Organizational climate relates to how employees perceive and describe the characteristics of their employing organization. It has been found to have an impact on healthcare professionals’ and patients’ experiences of healthcare (e.g. job satisfaction, patient satisfaction), as well as organizational outcomes (e.g. employee productivity). This research used organizational theory to explore dynamics between health care professionals (pharmacists, doctors and nurses) in mental health outpatients’ services for patients taking clozapine, and the perceived influence on patient care. *Setting* Seven clozapine clinics (from one NHS mental health Trust in the UK) which provided care for people with treatment resistant schizophrenia. *Methods* This study used qualitative methods to identify organizational climate factors such as deep structures, micro-climates and climates of conflict that might inhibit change and affect patient care. Using Interpretative Phenomenological Analysis, semistructured interviews were conducted with 10 healthcare professionals working in the clinics to explore their experiences of working in these clinics and the NHS mental health Trust the clinics were part of. *Main outcome measure* Health Care Professionals’ perceptions of the care of patients with treatment resistant schizophrenia. *Results* Three superordinate themes emerged from the data: philosophy of care, need for change and role ambiguity. Participants found it difficult to articulate what a philosophy of care was and in spite of expressing the need for change in the way the clinics were run, could not see how ‘changing things would work’. There was considerable role ambiguity with some ‘blurring of the boundaries between roles’. Factors associated with organizational climate (role conflict; job satisfaction) were inhibiting team working and preventing staff from identifying the patients’ health requirements and care delivery through innovation in skill mix. There were mixed attitudes towards the pharmacist’s inclusion as a team member. *Conclusions* Our findings suggest deficiencies within the clinics that may be manifestations of the wider culture of the NHS. The implications for mental health outpatient clinics are that local initiatives are crucial to the implementation of recovery models; clear guidance should be provided on the skill mix required in clozapine clinics and interprofessional learning should be encouraged to reduce role conflict.

## Impacts on practice

- The role and skill mix of multidisciplinary teams delivering clinical care should be better defined.
- Pharmacists can have a positive impact on the health of patients taking clozapine for treatment resistant schizophrenia.
- Pharmacists should be included in all clinical teams to ensure that patients’ medication needs are met.

## Introduction

Early research into organisational theory suggests the ‘atmosphere’, or ‘organisational climate (OC)’ in the workplace can affect personal and work outcomes [1]. The specific definition of OC has been the subject of debate for over half a century [2]. Burton et al. defined OC as ‘the attitude of the individuals [staff] concerning the organisation.’ (p. 69) [3]. The term has also been used interchangeably with organisational culture, defined by Schneider as “the shared basic assumptions, values, and beliefs that characterize a setting and are taught to newcomers as the proper way to think and feel.” (p. 362) [4]. In an attempt to provide clarity Ahmed [5] suggested that OC was what created organisational culture. Later authors proposed that climate is behaviourally oriented and is embedded in the organisation’s culture [6, 7] with Patterson et al. giving the example that ‘climates for safety or service represent the patterns of behaviour that support safety or service’ (p. 380) [7].

Prior research has examined OC and its relationship with employees’ perceptions of their job. In a study in a higher education institution (HEI), where employees were interviewed about their perceptions of their organisation [8], narratives revealed the impact that ‘deep structures’ such as power relations and management openness had on their perceived job security [8]. OC has also been studied in relation to job involvement, effort and performance [9]; job satisfaction [10, 11]; work attitudes and turnover [12]. In health care it has been linked with patient satisfaction [13], nurse turnover, absenteeism and injuries [14], nurses’ perceptions of their relationships with physicians [15], healthcare leadership [16] and evidence-based practice [17].

There may be more than one climate in an organisation, for example, Harborne and John [18] found a ‘micro-climate’ in more successful projects in the banking industry, regardless of the overall climate of the organisation. The micro-climate in this context is the atmosphere (OC) within teams working on particular projects. Within healthcare, local multidisciplinary team (MDT) delivery of care to people with complex and long term conditions is considered best practice and poor team cohesion has an adverse effect on both patient and professional outcomes [19]. Teams typically contain professionals from a variety of disciplines including doctors, pharmacists, nurses, physiotherapists and social workers, each with their own approaches towards patient care. Unless properly integrated, this diversity can reduce the effectiveness and efficiency of service delivery [20]. Where perceptions of staff and management do not match Malloy et al. suggest that ‘misunderstanding, under appreciation of roles and responsibilities and inconsistent decision-making’ (p. 720) may result [15]. The current study sought, through qualitative enquiry, to gain an insight into workplace factors that might be related to the climate of clozapine clinics.

### Aim of the study

The study reported in this paper explored the impact of OC on team working in climates prescribing clozapine to people with treatment resistant schizophrenia in one mental health Trust in the National Health Service in the UK. Such Trusts specialise in the treatment of patients with mental health problems through provision of in-patient and out-patient services. Specialist outpatient clozapine clinics are run because serious side effects can arise from treatment with clozapine, and patients require frequent and careful monitoring [21]. Individual staff perceptions of workplace procedures and policy were analysed as part of a larger mixed methods evaluation of the pharmacist’s role in clozapine provision [22].

### Ethical approval

A favourable ethical opinion was received from an NHS ethics committee specializing in research into mental health. Reference 08/WSE03/52.

## Method

Thirty health care professionals worked across seven clozapine clinics. The number of HCPs working in the area was small (n = 30) and their geographical remoteness made it difficult to study them as one group. Qualitative methods (one-to-one, semi-structured interviews, see Box 1), were chosen to capture an in-depth view of individual HCP perceptions of the influence of clinic organisation on the provision of care.

**Box 1:** Interview schedule content

*Interview topics*
1. Background/personal information
Can you tell me what services this clinic provides?
What do you think about these services for clozapine?
Do they work?
Do you think they are appropriate/sufficient?
2. Clozapine services: general
How and why was the service developed and how has it evolved, including institutional barriers and facilitators
Is there an underlying structure and philosophy of care in the clinic?
What services are provided?
How do these services support peoples’ recovery? i.e. what services are available and which do you think are most helpful?
How many professionals are involved in providing the clozapine services?
What role does each professional play within the clinic?
What do you think is a good configuration of professions to deliver clozapine services? Why?
3. Clozapine services: the users
How are people referred to the service?
What links are there with the persons’ general practitioner? How are they kept in the care loop/informed?
What other organisations or agencies does the clozapine team work with?
How do you share information across the organisation and with other disciplines?
Do you feel that you have enough time with each person to judge how well they are being supported? PROMPT clinically, emotionally, physically
Are you satisfied with the service that is provided?
If not how could it be improved?
Do you think the users of the service are satisfied?
4. Clozapine
What do you monitor in terms of outcome for clozapine? (PROMPTS e.g. clinical indicators, health protection and improvement. Social functioning, health and social care utilisation, patient experiences and satisfaction)
What information is given to clozapine users during their clinic attendance?
Does it change depending on their level of recovery?
How do you monitor compliance?
Is it a big issue for people?
How do you monitor side effects and which are the most important to monitor?
What about improvements in socialisation? PROMPT Re-integration into the community or getting back to work
5. Finally: Any other issues?
Is there anything else you would like to tell us about the way in which these services are provided?

All 30 HCPs were approached to take part in the research and 10 agreed to be interviewed—3 pharmacists, 3 doctors and 4 nurses. Nine interviews were carried out by one researcher (HF) and 1 was carried out by DAT, at the location of each participant’s choice. All participants chose the clinic in which they worked at a time when the clinic was quiet, in a place they could speak confidentially. Participants were informed that the interviews could take up to an hour but no time limit was imposed so they could talk for as long as they wished. Interviews were audio recorded, transcribed verbatim to ensure a faithful reproduction of the participants’ narratives and anonymised by the removal of identifying information. Transcripts were given an identifier e.g. Doctor 2, to enable comparison of the perceptions of the different HCP groups during the analysis stage. After each interview, HF and JS discussed each transcript and agreed on emerging themes that could be explored in later interviews. To ensure ‘credibility’ of the narratives, participants were offered the opportunity to see the transcription of their interview [23].

### Data analysis

Interpretative Phenomenological Analysis (IPA) [24] was used to enable an in-depth exploration of individuals’ experiences [23]. In line with the accepted process of analysis for IPA each transcript was coded line-by-line into themes using NVIVO-9 software. Similar themes from each transcript were brought together into groups and superordinate or ‘master’ themes were identified that exemplified the HCPs perceptions of the clinics and patient care. Similarities and differences amongst HCPs were compared and contrasted. The intention of IPA is to elicit meaning from participants’ narratives that are interpreted by the researchers [25]. Rather than counting the number of times each theme appears, it requires the researcher to actively engage with participants to make sense of what they are saying and to clarify and confirm meanings by a dialogue between participant and researcher. Trustworthiness of the data [26] was assessed by the coding of one transcript by both JS and HF. There was a high level of agreement regarding the codes and so JS continued with the coding and theming process. At the end of the coding process JS, HF and DAT met to discuss and clarify emerging themes.

## Results

The findings reported describe the participants’ perceptions of the provision of care. Three superordinate themes emerged from the data which were given titles by the researchers that represented the content of each theme. The themes that comprised each superordinate theme are shown in tabular form (Table 1). Extracts from participants’ narratives (shown indented in the text) were chosen as exemplars of experiences and attitudes amongst clinic staff and have been changed as little as possible to retain authenticity of the data. The participants’ unique identifiers are shown after each extract.

**Table 1:** Superordinate themes and themes

Philosophy of care	Need for change	Role ambiguity
Institutionalisation	Patients’ preferences	Blood testing
Recovery	Continuity of care	Knowledge of procedure
Patronising	Patient outcomes	Doctor’s view of pharmacists
Production line care	Mental state assessment	Role distinction
Attitude towards patients	Decreasing waiting time	Different perspectives
Deteriorating mental state = complaints	Effects of clozapine	Nurse’s perception of pharmacist input
Patient care priorities	Information for patients	Doctors’ lack of knowledge
Responsibility for patients	Waiting times	Nurse/pharmacist conflict
Paternalistic	Importance of routine for patients	No perceived role for pharmacist
Doctor’s perception of patient’s view	Patient satisfaction	Reason for no pharmacist input
Medicalisation	Better organisation of care	Role responsibility
	Prescribing skills	Blurring boundaries of job roles
	Better use of skills	Added value of pharmacists
	Pharmacists’ knowledge and skills	Doctor’s role
		Pharmacist presence
		Conflict with other HCPs
		Extension of job role

### Philosophy of care

A philosophy of care is a concept of care usually presented by an organisation as a core document which sets out the values that underpin that organisation’s service delivery. Such statements vary according to the type of care provided and help provide focus for members of the organisation. Many mental health services focus on recovery with the full involvement of HCPs and patients, families and carers. Without an understanding of the philosophy of care, staff cannot work together to achieve common goals. Themes such as ‘institutionalisation’, ‘recovery’, ‘patronising attitude towards patients’ and ‘production line care’ emerged from the participants’ narratives that showed a lack of awareness of the underlying principles of care. The following quotes exemplify the responses that were received from HCPs when asked if there was an underlying philosophy of care in the clinic (the use of the term was deliberate to see if the concept was understood). “I am sure there is one but I wouldn’t be able to state it categorically”. (D2).

and

Uhhh no. To offer good care in a nice way and friendly, say silly things and go ‘no more complaints’ [laughs] to family members [chuckling] …. No seriously, there might be but I don’t know where it is, and I don’t know what difference to my practice it would make even if there was one there because I just do it in a good way anyway. (N3)

The participants’ narratives then revealed more about attitudes towards patients. The quote from N3 illustrates an attitude amongst some staff that NHS patients with mental health conditions should not be making complaints. HCPs were also asked whether they felt the patients were satisfied with their care.

I’ve never asked them that question … because I always think that people that are not satisfied with the service - they wouldn’t come to it and they wouldn’t take the medication. The fact that they’re turning up regularly and we haven’t got to chase them for any reason……. But I would say they’ve never said that they’re not [satisfied], we’ve had clients in the past who have voiced that, but that was an indication of their mental state not being very stable rather than they didn’t like the actual service the clinic was providing. So I’ve never actually asked them, just presumed the fact that they turn up all the time indicates that they are. (N13)

A recovery model is based on the assumption that patients can remain in control of their lives following a period of mental ill health. One nurse was asked how staff supported patients’ recovery and she appeared not to understand the word ‘recovery’ asking if that meant ‘remission’. However, one of the doctors tried to explain it:

The recovery model is what the community psychiatric nurses should implement and the main purpose of the clinic is for them [the patients] to be safe on clozapine. The patients would have their own care co-ordinator who will look at the care plan and implementing that care plan in an holistic way. (D32)

The doctor above implied that recovery was something that nurses were responsible for and the role of the clozapine clinics was safe supply of medication. Although pharmacists would traditionally be associated with medicines use, some showed evidence of taking a more holistic approach. One pharmacist talked of activities that might help patients to recover from their condition and to become part of ‘normal’ life again.

Yes we have had people going into university doing courses …[pause] they often do voluntary work or yes, you know even if it is not paid it is something that they go out to do so it is something to get up for in the morning. (P5)

### Need for change

HCPs commented on the way the clinics were run suggesting a recognition that the provision of patient care was not ideal. Some felt that changes should be made if the clinics were to meet the needs of patients, but there was no sense of how change could be achieved. Further, staff seemed to be unaware that they themselves were in a position to generate the change process.

It’s not so much the staff side there’s things I’d like to see changed but it’s finding out how changing things would work - whether we could make those changes in practical terms and how to go about making those changes. (N8)

If changes were to be made there needed to be management support for staff initiatives. One pharmacist spoke of service improvement through better use of their skills as a prescriber, but the comment was underpinned by an acceptance of the attitudes inherent in the NHS.

With my prescribing I could have more time with the patients as well so that would be really good [pause]. You know sometimes they are just coming in and out quickly and you could give them a better service but you know it’s the NHS [laughs]. (P5)

There was a cynical view of the “management’s” knowledge of what clozapine clinics actually entail,

I don’t think higher up knows exactly what the service entails. I don’t know if that’s because they don’t know what I do, and they’ve been quite shocked about what I do and what I don’t do and they try and change things ‘cause they think I shouldn’t be doing what I do …but I think a lot of it as well is they don’t understand the issues. (N8)

Participants were asked about the potential for change. One of the pharmacists spoke of the lengthy waiting times some patients experienced when attending the clinics and suggested a change in the system.

I can see that they might have problems. They do have to wait a long time …sometimes people will push in front of other people because there is no proper system. Umm, I did think perhaps they could have a ticket system where they’re given a number when they come in so that, you know, they get their turn. There are odd things like the doctor doesn’t call the patients in so they sit around for sometimes a really long time waiting. (P7)

Some issues concerning change might be resolved if staff understood who was responsible for the clinics. There appeared to be no idea to whom staff were managerially accountable and the success of the clinics seemed to vary according to who was perceived to be in charge. Overall the exchange with this doctor (who was the identified clinical lead for all psychology and counselling services) exemplifies the response we received when we asked how the services were managed, “Well I am not sure, I think they report to umm, there is umm, obviously pharmacy input ….and umm people running those clinics will be line managed by which ever line manager would be managing them professionally”. (D32)

### Role ambiguity

All participants spoke of the need for clarification of the roles. There was an apparent lack of understanding of responsibilities within the team, and absence of agreement on the appropriate configuration of staff for the efficient running of the clinics. In particular, there were inconsistent views about the value of the pharmacists attending clinics. Their potential role was for monitoring the complex medication regimens of many patients and prescribing for the side effects of clozapine but one nurse did not perceive pharmacists as essential: “They’re [the pharmacists] involved with them [the clinics] but for those clinics that they’re not involved, in I don’t think those clinics are losing out on anything because they’ve still got access to pharmacy if they choose it”. (N8)

The doctors we spoke to seemed to have little understanding of the pharmacist’s role and were indifferent as to their involvement in the team. One doctor who had worked in a clinic with a pharmacist only realised (1 year on) that the pharmacist no longer worked there,

XX was able to prescribe and then if there were any queries that were beyond any of us XX could check them out umm so it was a very useful asset really. So actually it’s an interesting one - I haven’t really followed it up why we haven’t got one anymore, we haven’t had one for about a year. (D38)

Regarding the nurses’ views of doctors, one nurse was concerned that doctors assigned to the clinics were lacking specialist knowledge:

Some of the clinics haven’t got a doctor that knows anything about clozapine and I think if that’s the case sometimes you think ‘oh is it worth the doctor being there’ because they’ve got all the responsibilities but they haven’t got the knowledge to back it up. The patients are asking them questions and they haven’t got the answers or maybe they refer onto pharmacy. (N8)

One of the doctors agreed that for some clinics the doctor’s presence was simply a drain on resources.

I think a clinic which regularly has a medic involved can be quite a drain on medical resources…… I was expected to be at the clinic every day, every week and I would argue what my role was, because I was mostly taking bloods. (D2)

There was much discussion during the interviews about who should be drawing blood (required at every clinic visit to check for abnormalities before providing the clozapine). From the above extract the doctor did not think it was a good use of his time and other comments from pharmacists indicated that they felt it was the role of the nurse. One nurse, however, felt that as she was doing all the work, and should therefore make the decisions about the action that should be taken in the event of an abnormal result.

They all do it through me because I do the blood and it comes back through me and then I get a wodge of blood results and I go through and I check through them. But if there is anything obviously dangerous I have to do something about it straight away, errr and sometimes they [doctors] don’t agree with me anyway. (N3)

In the end it all appears to come down to what this doctor described as ‘blurring the boundaries’.

So I suppose you need someone to be able to take bloods, you need someone to be able to do mental state examinations and you need somebody who can prescribe and I suppose in this day ‘n’ age where you are blurring the boundaries between who can do that it is difficult to say, you know you just need people in those roles. (D2)

Although the comments made by the HCPs tended towards the negative aspects of team working, this final quote comes from a pharmacist prescriber who was responsible for the day-to-day running of one of the larger clinics. This pharmacist was positive about the benefits of team working and for this clinic it worked well.

We work as a team and we’ve all got our different skills. The doctor pops in if they want me to do anything, XXX is very chatty and knows all the patients really well and we just work well as a team. I think we catch a lot of people before if they become unwell. (P7)

## Discussion

OC has been described variously as the atmosphere in the workplace, perceptions of members of the organisation of its policies and procedures and attitudes of individual staff to the organisation. This study has reported an analysis of the climates within clozapine clinics in the British NHS. Interviews with a range of health care professionals indicated a lack of sense of direction, a recognition of problems, (but no ability (or inclination) to do anything about them), and ambiguity over roles, responsibilities and optimal team structures. The implication of the climate described by our participants was that HCPs did not work as part of a team with a common goal, or respect each other’s professions. With the OC in the workplace affecting personal and institutional outcomes [1], such negative perceptions auger poorly for patient outcomes and staff satisfaction. Although in 2005 the National Institute for Mental Health in England [27] endorsed a recovery model as a principle of mental healthcare, many of our participants showed either a lack of understanding of the concept or a perception that it was someone else’s job.

There is a sharp disparity between the apparent OC in the clinics that we studied and the rhetoric of the NHS which promotes a patient-centred and caring organisation [28]. Labelling complaining patients as ‘unwell’ is contrary to empowerment. In a national publically funded service such as the NHS, patients have no alternative provider, and it is especially important that their ‘voice’ should be able to be heard. The attitudes of our participants may be a manifestation of a ‘deep structure’ [8] of the NHS that is affecting staff perceptions and behaviours. The NHS is a huge organisation serving 54 million people, employing 1.6 million staff and incurring an annual cost (2015) of 116 billion British pounds [27]. Activity in the NHS is bound by an increasingly complex network of regulations and constrained by stringent spending limits. Policies are mostly determined remotely from the point of care, and unless handled sensitively, front line staff may perceive a lack of belonging or control that affects their everyday work and attitudes. Bakker and Schaufeli [29] suggest that organisations need ‘engaged workers’ who ‘take responsibility for their own personal development and who are committed to high quality performance standards’ (p. 147). Participants acknowledged that standards of patient care (such as waiting times, P7) could be better and expressed the need for changes in their individual clinics, but could not see how ‘changing things would work’ (N8). A study of clinicians and non-clinicians working in the Republic of Ireland [30] found some conflicting values and beliefs between the groups and a lack of trust. It attributes both positive and negative outcomes for the organisation, patients and staff to the values held, and suggests that HCPs should reconsider the values required for the delivery of healthcare.

A review conducted by Weaver et al. [31] indicates that patients are safer and receive a higher quality of care when that care is delivered by a team of HCPs. A large literature exists on the factors that promote effective interdisciplinary team working and strong leadership, managerial support, clear goals, good communication and clear standards for monitoring performance are agreed pre-requisites [32–34]. Deficiencies in such features underpinned the lack of understanding of own and others’ roles that our participants articulated. No clear consensus emerged about what skills were required and in what measure within the clinics. Innovations in medicines prescribing have resulted in nurses and pharmacists in the UK, USA and Australia being able to train to prescribe. The result has been that nurses and pharmacists have taken on roles once exclusive to the medical profession, bringing an end to medical dominance in this area [35]. Set in this context it is unsurprising that HCPs are no longer sure of what their roles entail in a clinic environment that is centred on medication provision, and climates of conflict result. There was a sense that nurses, pharmacists and doctors were trying to maintain a hold on their traditional roles but contemporary role changes made this harder to do, resulting in role ambiguity and conflict between staff members. It is in this context that leadership, guidelines and training for staff working as teams would be of benefit to both HCPs and patients.

Work conducted in Canada, on the integration of nurses into the MDT in a primary care setting [36] suggests that role clarification is both an organisational process and concerns professional competency. As HCP roles change with innovations in healthcare, training frameworks need to be adapted accordingly. Inter-professional learning (IPL) is now integrated into many training programmes for doctors, nurses and allied professionals to help them develop team working skills and prepare them for delivering high quality care in collaborative settings. Although doctors may tend to take the lead in treatment decision-making they have been shown to be willing and open to discuss options for care and to delegate to other professional groups [37]. In the context of clozapine clinics, where the main focus is on the safe prescribing of a medication with multiple potentially harmful side effects to a complex and vulnerable client group, enhancement of the role of pharmacists as clinic leaders is a potential option. Innovation in the delivery of care, however, depends on the incentives for reform in the local healthcare economy, and for success should involve attention to creating a physical, organisational and leadership environment that is conducive to team working. Such an OC will benefit patients, staff and the wider health service, and might be built on consultation, involvement and engagement of all staff in determining optimal team structures.

The main limitation of this study is that it is set in seven micro climates which may not be typical of others. Moreover, it was based on a small sample of healthcare professionals working in one NHS Trust. However, the qualitative nature of the study provides an insight into a specialist service that has the potential to prompt further reflection and study, particularly regarding the role of organisational issues in fostering a positive OC. Examples of well integrated MDTs, enabled by appropriate local organisational structures, are to be found throughout the NHS, and the study might have been strengthened if it had included an analysis of contrasting micro climates.

## Conclusions

Deficiencies within the micro climates that were the subject of this study may be manifestations of wider structural problems that can arise in a large and rigid organisation such as the NHS, and that require local initiatives to create productive and rewarding working environments. Since our study was conducted, a shared vision for integrated care and support has been launched by the NHS, which is underpinned by a national collaborative programme to help local organisations deliver it [38]. Guidance on collaborative working is widely available, the reach of IPL has increased and new models of commissioning are being trialled in efforts to ensure patients receive person-centred care. The nature of local responses will be crucial to facilitate micro climates in teams that ensure effective and efficient service delivery and enhance the satisfaction of patients and staff.
